# Dynamic Diagnostic Tests and Numerical Analysis of the Foundations for Turbine Sets

**DOI:** 10.3390/ma16041421

**Published:** 2023-02-08

**Authors:** Jerzy Szolomicki, Grzegorz Dmochowski, Maciej Roskosz

**Affiliations:** 1 Faculty of Civil Engineering, Wroclaw University of Science and Technology, 50-370 Wroclaw, Poland; 2 Faculty of Mechanical Engineering and Robotics, AGH University of Science and Technology, 30-059 Krakow, Poland

**Keywords:** machine foundations, FEM static and dynamic analysis, concrete testing, modelling structural elements

## Abstract

This paper shows current trends in testing and numerical analysis of dynamic loading in relation to a real frame foundation for a turbogenerator set. The analysis of the machine’s foundations, which are subjected to static and dynamic loads, is a complex problem combining the issues of geotechnics, structural engineering, and vibration theory. The authors present a case study of the assessment of the foundation’s technical condition. The main objective of this study is to perform and compare experimental and numerical dynamic analysis which includes the measurement of the acceleration, speed, and amplitude of the natural vibrations of the foundation during the operational speed of the turbogenerator. In addition, auxiliary material tests were carried out to fully diagnose the foundation and obtain the material properties required for the numerical analysis. They included both destructive and non-destructive of concrete strength, the evaluation of the degree of its carbonation, and the scanning of the reinforcement distribution. The research presented in the paper is intended to facilitate the preparation of appropriate data for the design of the foundation renovation and strengthening.

## 1. Introduction

The foundations of turbine sets are, in most cases, reinforced concrete supporting structures. The turbine sets consist of a synchronous generator that produces electricity, and a steam or gas turbine in which the enthalpy of the medium is transformed into mechanical energy of rotary motion [1]. In Poland, turbine sets are the most common energy sets that operate with the combustion of coal dust. The newer structures are gas or gas steam sets with a heat recovery steam generator. In such cases, the structure of the turbo set can be a multi-shaft and multi-body structure supported by metal foundation plates attached to a concrete foundation.

The shape of the foundation structure is mainly the result of the technical solution of the turbine set [2]. Foundations for turbine sets can be divided into two main types, depending on the shape of the structure: block foundations and frame foundations.

The block foundations are made up of a single-body reinforced concrete structure with numerous cutouts, which is placed on the ground or on piles. The basic feature of block foundations is their high stiffness, which allows them to be classified as non-deformable structures that are placed on elastic subsoil.

A variation of block foundations are:

- open box foundations,
- or closed box foundations.

The second type are frame foundations that consist of an upper plate or upper grate, columns or walls, and a raft plate set on the ground or on piles. The base raft plate, with a thickness selected to ensure the adequate stiffness and non-deformation of the entire foundation structure, is also intended to create conditions for the full restraint of the columns in the frame part. In frame foundations, vibrations are the result of the elasticity of the structure itself and, moreover, of the ground elasticity on which the bottom raft plate is placed.

Frame foundations are the basic type of supporting structure for high-speed machines that give lower inertia forces than reciprocating machines.

The main source of the dynamic forces in a turbine generator set are interactions that are related to the occurrence of vibrations with the frequency of the first and second harmonics and also those caused by its normal operation.

The loads from the turbine set to the foundation structure are transferred through the foundation bolts that fasten its individual parts. These bolts are screwed onto the recessed parts to the top plate of the foundation.

Foundations for turbine sets work mainly under dynamic load, and therefore high-quality materials with an appropriate strength and durability should be used for their construction. Due to the nature of the load and the need to maintain a rigid and homogeneous concrete structure with the same material properties, it is very important to ensure the homogeneity of the concrete in the entire foundation [3].

There are many scientific problems concerning the dynamics of foundations for turbine sets, which not only concern the structure itself, its geometric shape, material parameters, and boundary and initial conditions, but also those issues related to the identification of the place, value, and precise characteristics of the dynamic forces that originate from the rotating parts of the turbine set acting on the foundation structure [4,5]. The dynamic properties that characterise machines are the frequencies and forms of natural vibrations. In each rotor bearing (in the turbine and generator), the harmonic forces in the direction perpendicular to the longitudinal axis of rotation can be defined as:

(1) $$F_{i}\left(t\right)=P_{i}\sin\left(\omega t\right),$$

(2) $$P_{i}=m_{i}e\omega^{2},$$

where:

*m_i_*—the proportional part of the rotating mass supported by the *i*-th bearing,
  *e*—the eccentricity of the mass,
  ω=2πf—the cyclic frequency of the turbogenerator’s operation,
  *f*—the operating frequency (*f*_0_ = 50 Hz at the nominal speed of the turbine).

The harmonic analysis of the foundation is performed for frequencies in the range of 0.8 *f*_0_ ≤ *f* ≤ 1.2 *f*_0_ between 40 and 60 Hz [6].

**Figure 1:**
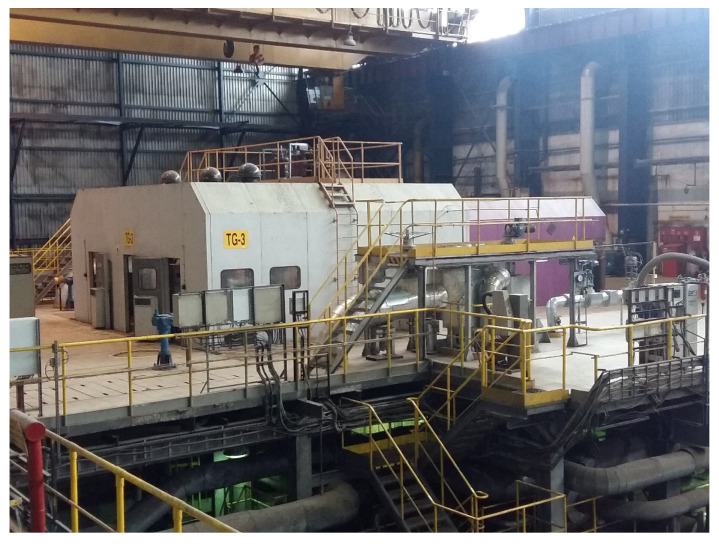
General view of a turbogenerator.

Diagnostics of the structure of foundations for a turbogenerator (Figure 1) is one of the most complicated [7,8,9]. It includes tests, calculations, and analyses that take into account the actual state of the structure and the occurring loads and operational factors, and which also require the adoption of an adequate model that shows the behaviour of the structure [10,11]. Therefore, structural diagnostics often require the use of the most modern research methods, as well as complex variants of analytical methods that use elaborate computer simulations [12,13,14,15,16]. The main objective of this study is to perform and compare experimental and numerical dynamic analysis which includes the measurement of the acceleration, speed, and amplitude of the natural vibrations of the foundation during the operational speed of the turbogenerator. In addition, verification of resonance conditions and weather vibration magnitude must satisfy code limits. Results of the experimental tests in situ enable one to design foundation structure strengthening and update structural models for numerical analysis. The research presented in the paper is intended to facilitate the preparation of appropriate data for the design of the renovation and strengthening of the foundation.

## 2. Materials and Methods

The authors analysed the existing turbogenerator foundation for a combined heat and power plant. The following experimental dynamic tests were used to verify the resonance conditions and magnitude of vibrations:

- measurement of the acceleration, speed, and natural vibration amplitude of the foundation during the normal operation of the turbogenerator.

**Figure 2:**
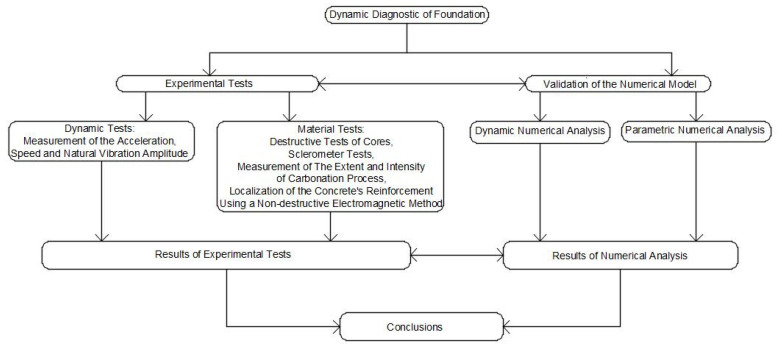
Flow chart of the analysis performed.

In addition, the following material tests were carried out to fully diagnose the foundation and obtain the material properties required for the numerical analysis:

- destructive tests of cores cut from the structure in order to assess the compressive strength of concrete;
- investigations concerning the homogeneity of strength characteristics, and the estimation of the concrete’s strength grade on the basis of sclerometer tests [17,18];
- measurements of the extent and intensity of the carbonation process of the subsurface concrete layer conducted on the cut cores using the rainbow test [19,20];
- localisation of the concrete’s reinforcement and the determination of its arrangement, its diameters, and the thickness of the concrete’s cover using a non-destructive electromagnetic method [21], followed by the comparison of the obtained results with archival documentation. Figure 2 shows a general flow chart of the analysis performed.

The experimental tests were carried out with direct access to the structure and the use of test equipment that met all the necessary technical requirements for this type of measurement. After the experimental analysis of the foundation, the next step of research was numerical modelling with parametric study. It included its structure, which was considered as a reinforced concrete frame system founded on the ground through the raft plate. Dynamic numerical analysis was carried out using AxisVM X6 software. The calculation of the natural frequencies and the appropriate mode shapes is the basis for determining the dynamic parameters of the foundation. The numerical determination of the eigenfrequencies and the form of vibrations of the foundation TG is quite difficult in this case and, therefore, was verified by experimental measurements. Results of experimental analysis were utilised for creating a numerical model.

## 3. Dynamic Actions—A Literature Review

Dynamic actions are additional loads on the foundation that must be taken into account in strength calculations. In the dynamic analysis of the foundation, the natural frequencies are determined (there should be a 20% difference compared to the operating frequency of the machine [22]) to check the possibility of the occurrence of resonance. It is also checked if the vibration amplitude resulting from the operation of the turbogenerator is within the acceptable standard limits. In addition, the calculations include the determination of stresses in various structural elements of the foundation (columns, beams, top plate, and base raft plate in the case of a frame foundation) and the checking of their load-bearing capacity.

The scientific literature contains many research results that concern a wide spectrum of machine foundation analysis. The main purpose of the research is to identify the dynamic parameters that are extremely important in the diagnosis of this type of structure. Among the various analyses available in the literature, the following can be distinguished:

- research on the interaction between the foundation and the soil with the determination of the displacements and internal forces of the foundation using three-dimensional viscoelastic boundary elements for the model of the upper plate of the frame foundation [23];
- estimating the foundation parameters in the event of failure, using the inherent unbalance of the rotor and developments in modelling for improved balancing [24,25,26];
- simulation analysis for asynchronous operation capacity of the turbogenerator under excitation loss [27];
- study of the superposition of vibrations and analysis of ground sensitivity [16];
- numerical analysis with the use of FEM programs (ANSYS, SAP, STAAD) in order to carry out a modal analysis to determine the frequency and amplitude of the vibrations of the foundation [28,29,30];
- studies of the effects of seismic interactions and the structural configuration for the natural vibrational frequencies of the structures and seismic resistance estimation of existing turbogenerator foundation by a non-linear static method [31,32];
- field tests of frame foundations in terms of settlement and resistance to temperature load [33];
- an investigation of the influence of the supporting structure on the dynamics of the rotor system [34]
- an investigation of stiffness, damping value, natural frequencies, and vibration mode shapes by modelling the soil–foundation system using the FEM [35,36,37,38,39];
- tests of the damping coefficient conducted on the basis of the analysis of the measuring signal using the wavelet transform [40,41];
- analysis of multi-criteria optimisation with regards to the foundations for the turbogenerators [42,43];
- dynamic analysis of a thin and narrow turbogenerator foundation on piles with differentiation of frequency, shear wave velocity, and mode shape [44] and determination procedure of load-bearing capacity [45];
- the estimation of multiple fault parameters of a fully assembled turbogenerator system based on the least squares technique requires forced response information [46].

## 4. Case Study—A Foundation for a Turbogenerator in a Combined Heat and Power Plant

This paper analyses an existing foundation for a turbogenerator directly coupled to a steam turbine with a rotational speed of 3000 rpm. The turbine is an axial single body and has four vents and two steam outlets for heaters. Steam from the first vent is taken for technological and heating purposes, and from the remaining three, it is directed to low-pressure regenerative exchangers. The turbine set is supported by three bearings, one of which is the load bearing.

**Figure 3:**
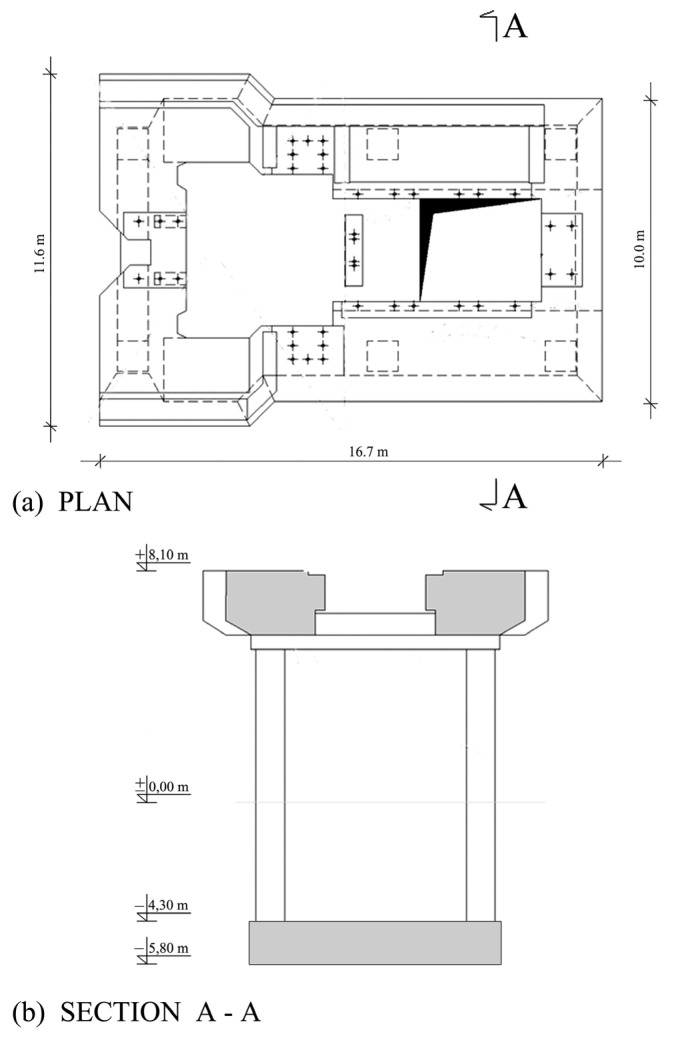
Foundation structure: (**a**) plan; (**b**) section.

The analysed foundation is a reinforced concrete frame structure that consists of a raft plate, columns, and a top plate, as shown in Figure 3. The raft plate of the foundation is set directly on the ground. The part of the reinforced concrete frame of the foundation is supported on this plate. The top plate is supported by three pairs of columns. The basic thickness of the top plate in the generator section is 2.50 m. All columns have cross-sectional dimensions equal to 1000 mm × 1000 mm.

**Figure 4:**
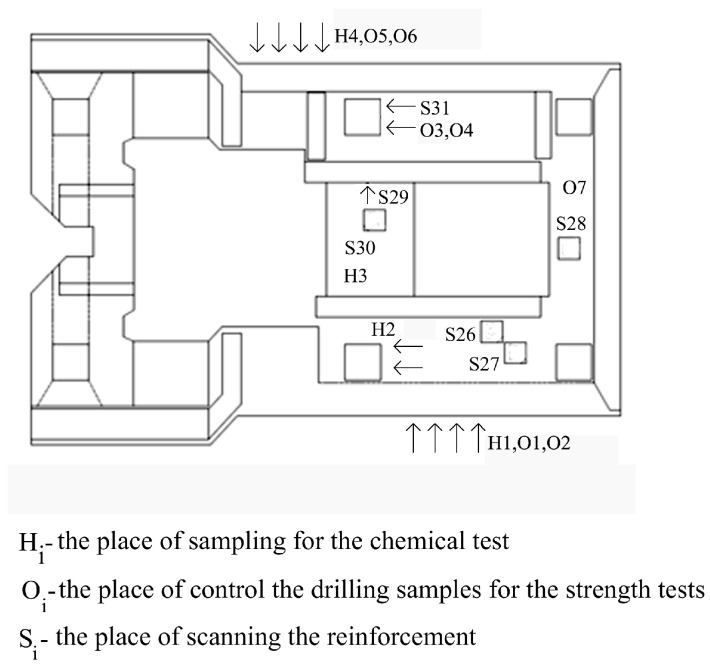
Localisation of measurement sites on the surface of the tested foundation.

To diagnose the foundation (Figure 4), dynamic and material and numerical analyses using the results of the experimental tests were carried out.

### 4.1. Dynamic Experimental Test

Dynamic tests were performed with impulse forced vibration of the foundation structure to determine the basic foundation parameters.

#### Measurement of the Amplitude of Foundation Forced Vibrations

**Figure 5:**
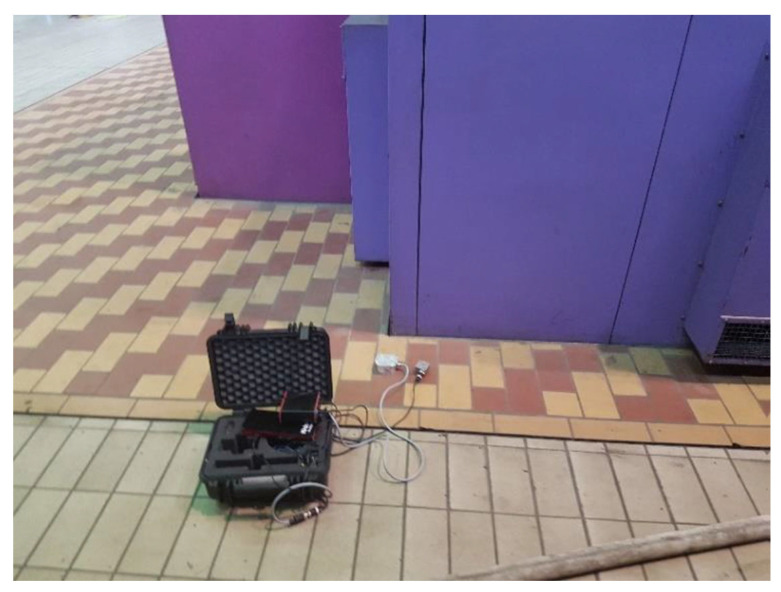
Measurement of vibrations at the operating speed of the turbine placed on the top plate of the foundation using a piezoelectric accelerometer.

**Figure 6:**
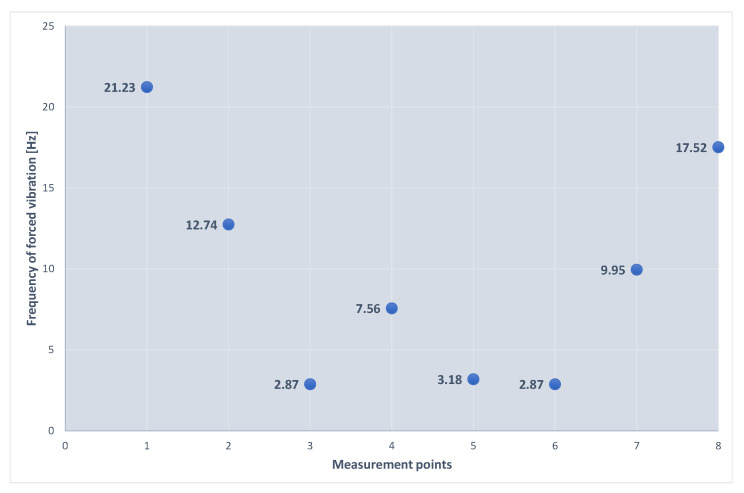
Forced vibration frequency diagram for individual measurements points.

At the level of the floor slab, the measurements of vibrations at the operating speed of the turbine set were conducted in eight points of the foundation’s top plate using a piezoelectric accelerometer (Figure 5). As a result of the research, the following root mean square (RMS) values of the acceleration of vibrations, the velocity of vibrations, and the displacement of the foundation are presented in Table 1. Figure 6 illustrates the frequencies of forced vibrations determined on the basis of the measurements. The measured values of the average displacement amplitudes are between 1 and 5 μm, which is much lower than the permissible amplitude for the foundations of turbogenerators.

### 4.2. Auxiliary Material Experimental Tests

#### 4.2.1. Assessment of the Compressive Strength of Concrete

**Figure 7:**
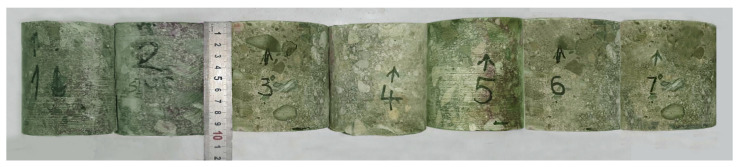
The control drilling samples cut from the tested fragments of the foundation structure.

The compressive strength of concrete was determined on the basis of core samples ϕ × h = 100 × 100 mm that were cut from the elements of the tested structure, as shown in Figure 7. The measurement site was previously scanned with a reinforcement detector to avoid cutting the rebars when drilling the core samples. The test was carried out with a BOSCH drilling set in accordance with the procedures specified in standards EN 12504:1:2009 and EN 12390-3:2009.

The sampled drillings were compressed in a testing machine. The strength grade of the concrete was determined on the basis of the results of the destructive tests of the core samples (Table 2). It was determined in relation to standard EN 13791:2019-12.

As a result of the calculations, the following were obtained:

- the average value of the concrete’s compressive strength $f_{m\left(n\right),is}=35.06\;\mathrm{MPa}$,
- the minimum value of the concrete’s compressive strength $f_{is,lowest}=29.5\;\mathrm{MPa}$,
- standard deviation $s_{R}=4.34$.

According to the standard EN 13791:2019-12, it was assumed that the characteristic strength of concrete in the tested elements is the lower of the following two values:

(3) $$f_{ck,is,cube}=f_{m\left(n\right),is}-k_{n}*s\;\mathrm{or}\;f_{ck,is,cube}=f_{is,lowest}+M$$

where:

$f_{ck,is,cube}$—characteristic compressive strength of the concrete in the structure, which corresponds to the strength of the concrete determined on cubic samples with a side length of 150 mm;
$f_{m\left(n\right),is}$—the average value of the concrete’s compressive strength in the structure obtained from n measurement results;
$f_{is,lowest}$—the lowest of the determined values of the compressive strength of the concrete in the structure;
  *k_n_*—coefficient that depends on the number of samples *n* = 7, *k* = 2.

After the calculation, the following was obtained:

(4) $$f_{ck,is,cube}=35.06-2\times 4.34=26.38\;\mathrm{MPa}$$

or

(5) $$f_{ck,is,cube}=29.5+4=33.50\;\mathrm{MPa}$$

Based on the results of the tests obtained, it can be assumed that the value of the characteristic strength of the concrete tested in the foundation structure is not higher than 26.38 MPa and, according to the EN 13791:2019-12 standard, it is assumed that its strength grade corresponds to the archival documentation.

#### 4.2.2. Sclerometer Test of Concrete

Concrete homogeneity tests were carried out using a Schmidt “N” sclerometer according to the procedures specified in the standard PN-EN 12504-2:2021-12. Measurement locations were assumed in the following foundation elements: a horizontal longitudinal beam, an outer column, and a middle column. The results of the sclerometer measurements presented in Table 3 and Table 4 were correlated at the drilling sites. Sclerometer measurements were used to assess the quality of the concrete on the basis of the homogeneity of its strength properties.

(6) $$L_{av}=\sum L_{i\;}/n=48.22\;\mathrm{MPa}$$

(7) $$s_{L}=\sqrt{\frac{\sum {\left(L_{i}-L_{av}\right)}^{2}}{n-1}}=2.06\;\mathrm{MPa}$$

(8) $$n_{L}=\frac{s_{L}}{L_{av}}\cdot 100\%=4.28\%$$

The following equation was adopted as the hypothetical regression equation:

(9) $$R_{av}=L_{av}\cdot (0.0356*L_{av}*\left({\left(\frac{n_{L}}{100}\right)}^{2}+1\right)-0.795+\frac{6.4}{L_{av}}=51.00\;\mathrm{MPa}$$

(10) $$\begin{array}{c} s_{R}=L_{av}\cdot \left(\frac{n_{L}}{100}\right)\cdot \sqrt{0.00254\cdot L_{av}^{2}\cdot ({\left(\frac{n_{L}}{100}\right)}^{2}+2)-0.1134\cdot L_{av}+0.633}=5.45\;\mathrm{MPa} \end{array}$$

(11) $$n_{R}=\frac{s_{R}}{R_{śr}}\cdot 100\%=10.69\%$$

(12) $$R_{min}=R_{śr}-1.64\cdot s_{R}=42.06\;\mathrm{MPa}$$

Correction coefficients that depend on the concrete age *α* = 0.6 and the dry air state *β* = 1 were adopted in the analysis.

(13) $$R_{b}^{G}=\alpha \cdot \beta \cdot R_{min}=25.24\;\mathrm{MPa}\;$$

(14) $$L_{av}=\sum L_{i\;}/n=52.13\;\mathrm{MPa}$$

(15) $$s_{L}=\sqrt{\frac{\sum {\left(L_{i}-L_{av}\right)}^{2}}{n-1}}=0.90\;\mathrm{MPa}$$

(16) $$n_{L}=\frac{s_{L}}{L_{av}}\cdot 100\%=1.72\%$$

The following equation was adopted as the hypothetical regression equation:

(17) $$R_{av}=L_{av}\cdot (0.0356*L_{av}*\left({\left(\frac{n_{L}}{100}\right)}^{2}+1\right)-0.795+\frac{6.4}{L_{av}}=61.73\;\mathrm{MPa}$$

(18) $$\begin{array}{c} s_{R}=L_{av}\cdot \left(\frac{n_{L}}{100}\right)\cdot \sqrt{0.00254\cdot L_{av}^{2}\cdot ({\left(\frac{n_{L}}{100}\right)}^{2}+2)-0.1134\cdot L_{av}+0.633}=2.62\;\mathrm{MPa} \end{array}$$

(19) $$n_{R}=\frac{s_{R}}{R_{śr}}\cdot 100\%=4.25\%$$

(20) $$R_{min}=R_{śr}-1.64\cdot s_{R}=57.43\;\mathrm{MPa}$$

The analysis adopted correction coefficients that depend on the concrete age *α* = 0.6 and the dry air state *β* = 1. Finally, the guaranteed concrete strength is:

(21) $$R_{b}^{G}=\alpha \cdot \beta \cdot R_{min}=34.46\;\mathrm{MPa}$$

Based on the sclerometer tests, the concrete strength grade in the horizontal beams was estimated as C20/25 and in the columns as C25/C30. Finally, concrete was adopted for the entire structure according to the C20/C25 class. It is one class weaker than was assumed in the archival project.

#### 4.2.3. Measurement of the Intensity of the Carbonation Process of the Subsurface Concrete Layer

**Figure 8:**
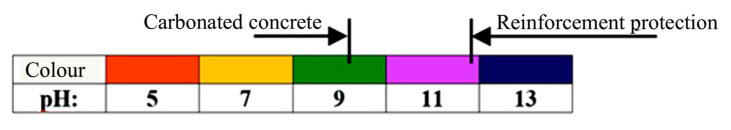
A colour template for assessing the depth and intensity of the carbonation process for the rainbow test.

Under the influence of the carbon dioxide contained in the atmosphere (${\mathrm{CO}}_{2})$, and the moisture in the pores of the concrete, the subsurface concrete layer undergoes a gradual process of carbonation. The carbonation front gradually moves deeper into the concrete, with the main reaction in this process being the reaction of carbon dioxide with calcium hydroxide. As a result of this reaction, calcium carbonate $\left({\mathrm{CaCO}}_{3}\right)$ is formed. This lowers the reaction of the concrete, which in turn leads to a gradual loss of the protective properties of the concrete against steel. The pH of fresh concrete is 11.8–12.6. It is assumed that a decrease in the concrete’s pH to about 10.0–11.8 causes the loss of stability of the protective passive layer on the steel. Within the performed research, the scope and intensity of the carbonation process were assessed using the rainbow test. This test allows the pH distribution profile to be determined within the range of 5.0–13.0 (with gradation every two pH degrees). Measurement involved spraying the surface of the fresh fracture of the tested element with the indicator and then determining the pH distribution based on the colour table, as shown in Figure 8. The tests were carried out according to the procedures specified in the standard PN-EN 12390-12:2020-06.

**Figure 9:**
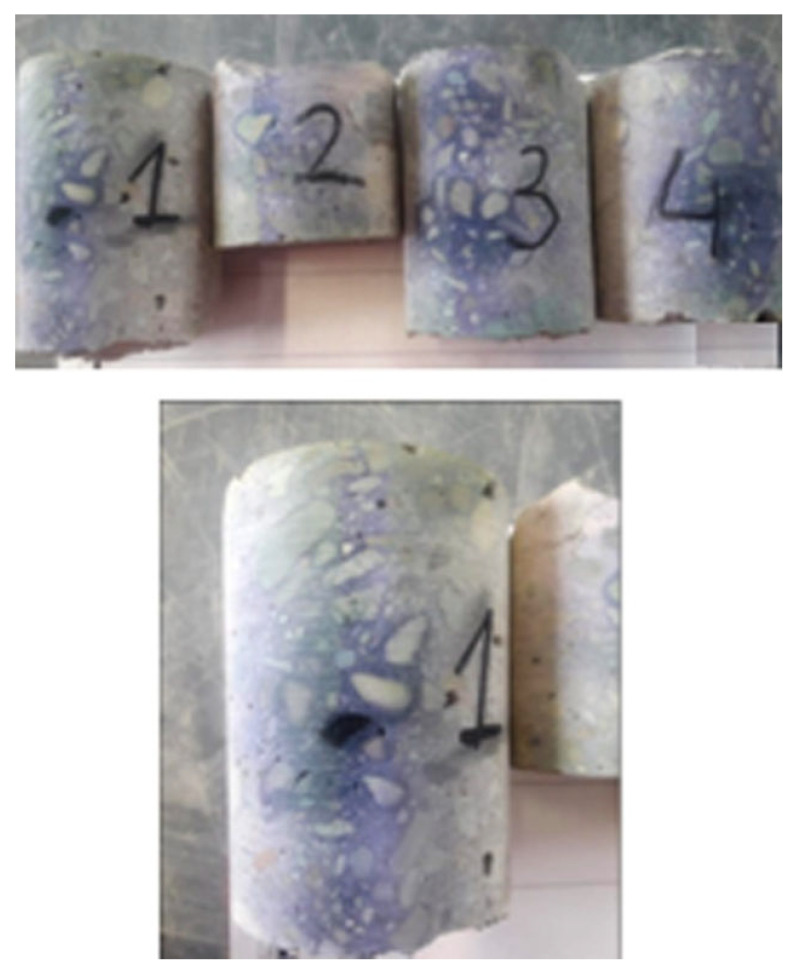
Test of the extension of concrete carbonation.

Measurement of the carbonation intensity was carried out on four drilling core samples from the foundation columns and beams, as shown in Figure 9. No carbonation was found in these samples. The main reinforcement bars have a cover of 2.5–3.0 cm. No corrosion was found in the uncovering of the main reinforcement (made of Ø32 bars) in either the beams or columns.

#### 4.2.4. Investigation of the Thickness of the Concrete’s Cover and the Location and Diameter of the Reinforcement

**Figure 10:**
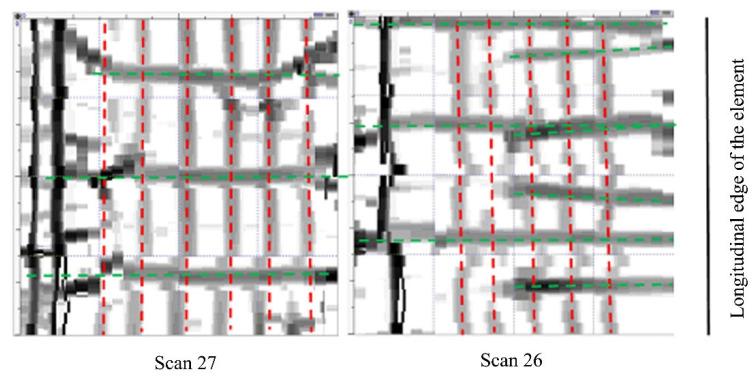
The image of reinforcement scanning at the S26 and S27 measurement sites (at the bottom of the foundation body); red means bars of the main reinforcement; green means cross-bars.

**Figure 11:**
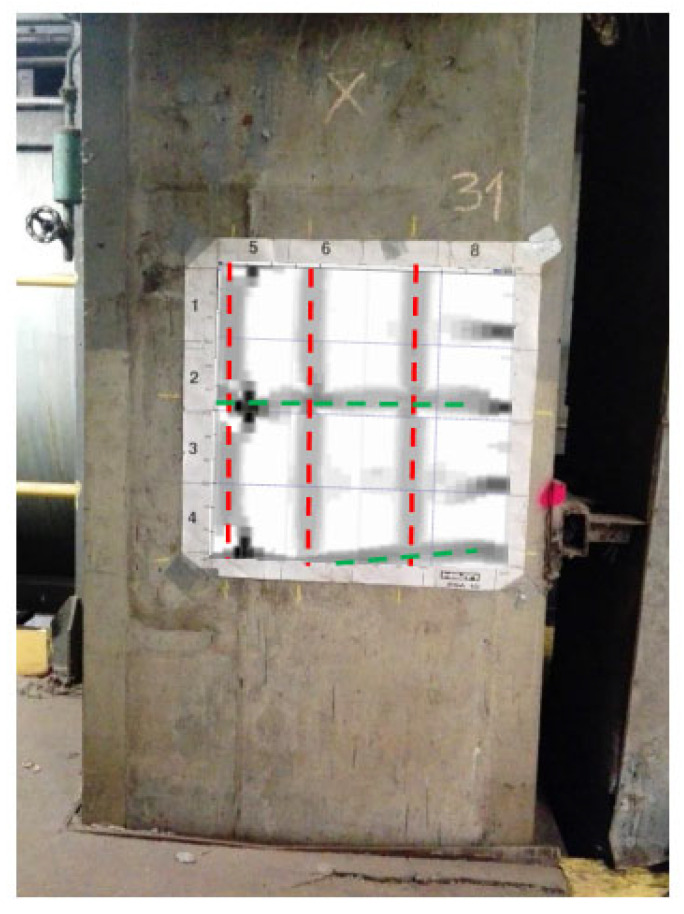
The image of reinforcement scanning at the S31 measurement site (column area under the foundation body) superimposed on the tested element.

Measurements were carried out in a non-destructive manner with the use of specialised Hilti Ferroscan instruments that are intended for locating the reinforcing bars and for measuring the thickness of the concrete’s cover [47]. The measurements used the electromagnetic method of excitation of currents in the reinforcement. The instruments automatically calculate the cover thickness as the smallest distance between the bar side and the concrete surface for a given bar diameter. The electromagnetic method is a research method that uses the phenomenon of induction of a current in an electric circuit that is caused by the electromagnetic field of the circuit being disturbed. Testings of reinforced concrete structures with the use of the electromagnetic method involve successive scanning of concrete surfaces with a measuring probe to locate the reinforcing bars, followed by the determination of their diameter and thickness. Before measuring the thickness of the cover, the diameters of the reference bars, determined on the basis of the technical documentation or during a micro-uncovering, are entered into the device. The tests were carried out at six foundation measurement sites (S26–S31) on an area of 60 cm × 60 cm, as shown in Figure 10 and Figure 11.

The scanning of the reinforcing bars confirmed that their actual distribution is similar to that presented in the technical documentation. On the basis of the performed uncoverings, it was found, in the case of the columns and beams, that the main reinforcement is consistent with the archival documentation, has a diameter of 32, is made of 18G2 steel, and that the stirrup spacing in the beams is equal to 20 cm.

### 4.3. Numerical Analysis of the Foundation

**Figure 12:**
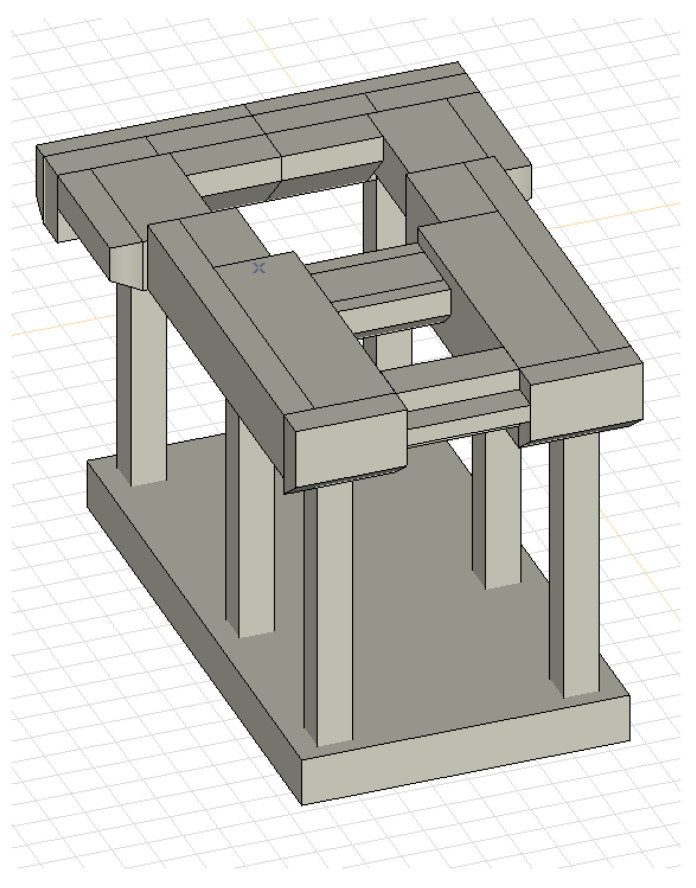
Numerical FEA model of the foundation’s geometry.

**Figure 13:**
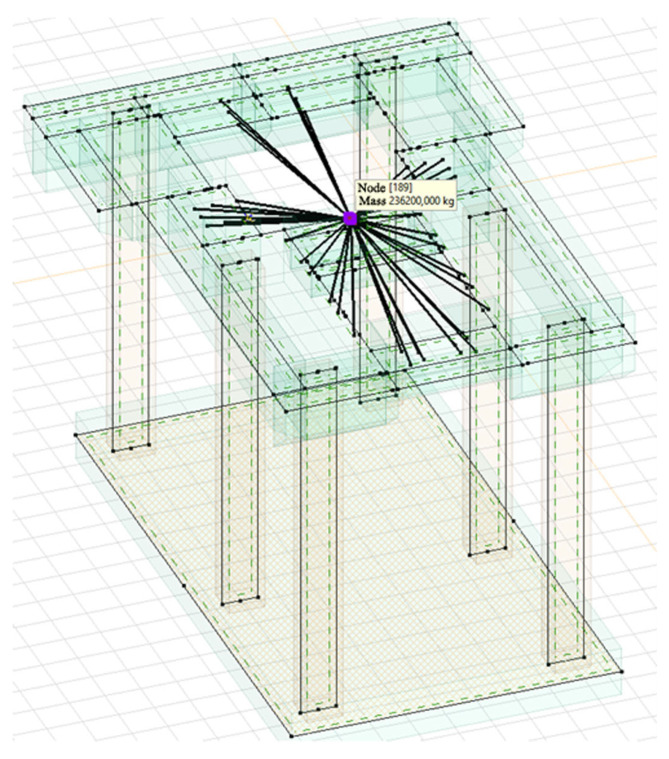
FEA model of the foundation’s load.

On the basis of the experimental tests and the archival technical documentation, a numerical analysis was performed using the FEM, as shown in Figure 12. The analysis involved the verification of the conditions of the ultimate and serviceability limit states of the foundation, and the performing of tests of forced vibrations. Dynamic numerical analysis was carried out using AxisVM software. The permanent load was defined as the self-weight of the foundation’s structure and the weight of the turbine set. In the numerical model, it was modelled as a concentrated mass that is connected, using rigid elements, to the turbine set’s fastening points in the foundation, as can be seen in Figure 13. The analysis assumed an elastic ground, the characteristics of which were determined on the basis of archival research.

The self-weight was determined on the basis of the foundation’s dimensions, while the weight of the turbine set was assumed according to the technical documentation. For a generator with a stator, the weight is 169.5 t, and for a turbine, it is 66.7 t.

#### 4.3.1. Analysis of the Amplitude of the Foundation’s Vibration

In the dynamic calculations, the load from misalignment of the rotating parts of the turbogenerator, i.e., the stator rotor and the turbine rotor, was taken into account. This load is assumed based on the equation:

(22) $$F_{s}=M_{w}\cdot g\cdot n\cdot 0.2$$

where:

M_W_—the mass of the rotating part: stator rotor: 30,150 kg, turbine rotor 17,000 kg,
  g—acceleration due to gravity,
  n = 1.2.
  Finally assumed:
  $F_{s,\mathrm{stator}}=70.96\;\mathrm{kN}$,
  $F_{s,\mathrm{rotor}}=40.34\;\mathrm{kN}$.

On the basis of the technical documentation, it was assumed that the foundation raft plate is set on subsoil that consists of gravel mix with the degree of compaction $I_{D}=0.5$. Therefore, the following elasticity coefficients of the subsoil were adopted for foundations with an area greater than 50 m^2^:

$$C_{z}=50\;\mathrm{MPa}/m,$$

$${\;C}_{x}=C_{y}=0.7\cdot C_{z}=35\;\mathrm{MPa}/m.$$

**Figure 14:**
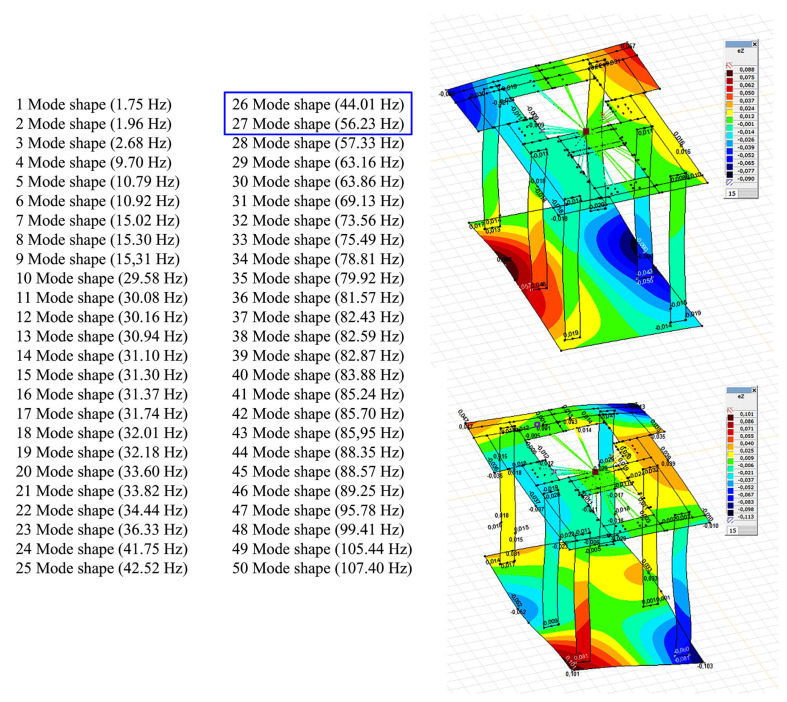
Calculation results of the foundation’s natural frequency obtained by AxisVM software: (**a**) amplitude of foundation displacement for 26 mode shapes of frequency (44.01 Hz); (**b**) amplitude of foundation displacement for 27 mode shapes of frequency (56.23 Hz).

**Figure 15:**
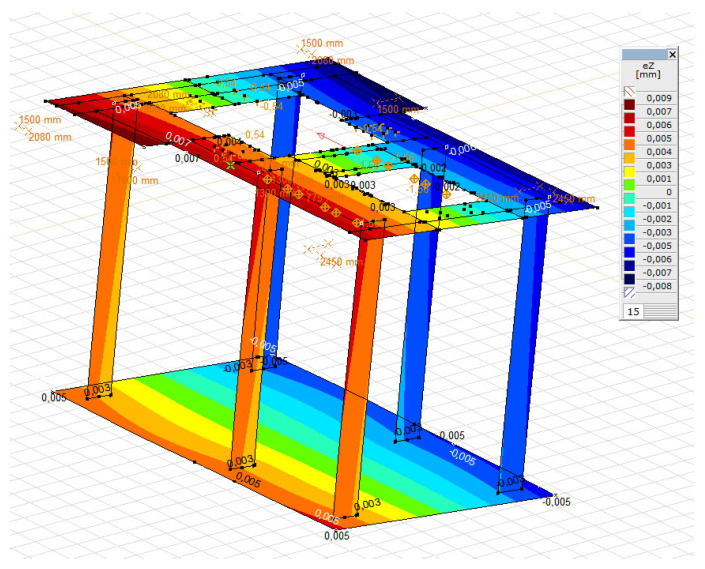
Maximum amplitude of foundation displacement for forced vertical vibrations obtained by the AxisVM software.

The calculated natural frequencies of the foundation do not indicate the presence of a resonance state with any mode shape, as shown in Figure 14. The natural frequencies obtained, which are closest to the frequency of the reactive force, are the frequencies for the 26 and 27 mode shapes (44.01 Hz, 56.23 Hz). The exciting forces were applied at the places where the rotor and stator are fastened. For the load scheme adopted in this way, the forced vibration amplitudes were calculated for individual values of transient resonances. The amplitude of the vertical forced vibrations of the top foundation plate, both at the operating rotational speed of the machine and during transient resonances, does not exceed 9 μm (Figure 15). This is much lower than the permissible vibration amplitude for turbogenerator foundations, which is 20 μm [48]. The calculated theoretical vibration amplitude of the foundation corresponds well with the actual measured vibration amplitude of the foundation during normal operation of the turbogenerator, which is equal to 3 µm (the measured vibration amplitude was classified as not degrading the foundation).

#### 4.3.2. Analysis of the Load-Bearing Capacity of the Foundation

The EN 1991-3 standard was used to calculate the dynamic forces caused by rotation. The interaction effect which results from the excitation of the machine with the rotating masses and the dynamic behaviour of the structure can be expressed by an equivalent static force defined as:

(23) $$F_{\mathrm{eg}}=F_{s}\cdot \varphi_{M},$$

where:

F_s_—the centrifugal force of the rotating part equal to 111.3 kN,
  $\varphi_{M}$—the dynamic coefficient which depends on the ratio of the natural frequency n_e_ to the frequency of the excitation forces n_s_:

(24) $$\varphi_{M}=\left[{\left(1-\frac{n_{s}^{2}}{n_{e}^{2}}\right)}^{2}+{\left(\frac{2\varsigma n_{s}}{n_{e}}\right)}^{2}\right]{,}^{0.5}$$

where:

ζ—the damping coefficient, n_s_ = 50 Hz—frequency of the exciting force.

According to [24], for turbogenerators on RC frame foundations, the damping coefficient is defined as:

(25) ζ= Δ/2π,

where:

Δ—the logarithmic damping decrement of the foundation, which is equal to approx. 0.4 for RC frame foundations.
  After inserting n_e_ = 44.01 Hz,
  φ_M1_ = 3.1.
  After inserting n_e_ = 56.23 Hz,
  φ_M2_ = 4.2.
  Finally, the following was assumed:
  φ_M_ = 4.2.

According to [24], the computational value of the forces, which replaces the impact of dynamic loads on the foundation, is obtained from the following formula:

(26) $$P_{d}=\;\varphi M\;µ\;\gamma \;\mathrm{Pc}$$

where:

$\varphi_{M}$—dynamic coefficient (as above),
  µ—fatigue factor equal to 2,
  γ—calculation factor equal to 5.

The centrifugal force of a rotating part F_s_ = 111.3 kN.

For the purposes of the calculations, this force was divided into the force from the stator rotor and the turbine rotor:

F_s,stator_ = 70.96 kN, adjusted to the value of 71.0 kN,
  F_s,turbine_ = 40.34 kN, adjusted to a value of 40.3 kN.

The design value is as follows:

F_s,stator,eq_ = 4.2 · 2 · 5 · 71 = 2982 kN,
  F_s,turbine,eq_ = 4.2 · 2 · 5 · 40.3 = 1693 kN.

The forces determined in this way were loaded on the turbogenerator’s foundation. Additionally, the exceptional moment derived from the start up and stop run up loads was considered in the calculations. According to the EN 1991-3 standard, for this moment, the equivalent static moment can be calculated in the following way:

(27) Mk,eq =1.7 Mk,max,

where:

M_k,max_—the peak value of the moment derived from the start up and stop run up loads according to the archival documentation:

(28) Mk,max = Fzw · a,

where:

F_zw_ = 876.3 kN—start up and stop run up load,
  a = 3.69 m (spacing of fastening bolts),
  M_k,max_ = 3233.55 kNm,
  M_k,eq_ = 1.7·3233.55 = 5497.03 kNm.

**Figure 16:**
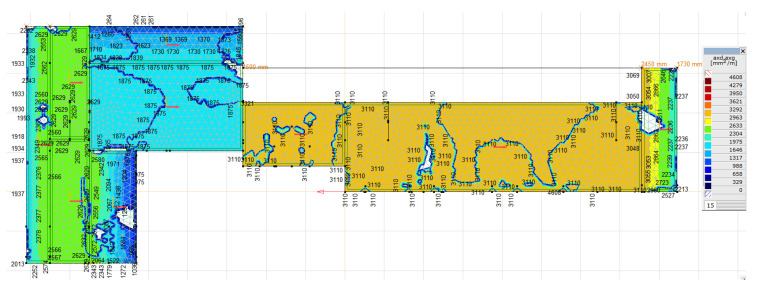
Bottom and top reinforcement of the top plate in the horizontal direction (main direction) obtained by the AxisVM software.

**Figure 17:**
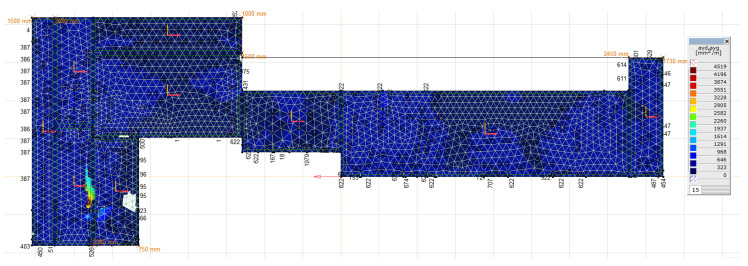
Bottom and top reinforcement of the top plate in the vertical direction (main direction) obtained by the AxisVM software.

For such loads, the required main reinforcement was calculated, as can be seen in Figure 16 and Figure 17. The obtained values of the reinforcement area do not exceed the value of the reinforcement applied in the foundation, that is, min. 5 × ϕ 32 per 1 m = 4020 mm^2^/m. The execution of the numerical calculations of the natural frequencies and mode shapes of the foundation construction was possible thanks to the research and measurements conducted. The theoretical vibration amplitude of the foundation is greater than that measured during normal operation of the turbogenerator; however, both are lower than the permissible value. The calculated values of the stresses in the concrete and reinforcing bars are lower than the permissible values.

## 5. Discussion

The performed dynamic analyses show the great possibilities of using the FEM in the diagnostic process of foundation structures for turbogenerators. Due to the enormous cost of such objects, the use of FEM allows one to accurately verify the entire structure not only for the dynamic behaviour of the object but also for the structural strength and resistance to earthquake-type excitations. The complexity of such an object requires a very accurate reconstruction of the whole structure, together with taking into account the correct material parameters and dynamic characteristics. Therefore, the creation of a proper numerical model required conducting experimental dynamic and material tests of the foundation. The results of the experimental tests, especially in situ, enable the design of the existing foundation structure strengthening for future performance and update the initial foundation structural model for final numerical analysis. It can be stated that numerical analysis allows for better recognition of foundation properties, dynamic damping characteristics, fatigue, rheological changes, corrosion, and the degree of efficiency due to exploitation. The research presented in this paper was based on a limited amount of data. This problem, which affects the effectiveness of numerical analysis, can be solved in the future by using artificial neural networks. The ANN represents an artificial system based on mathematical models similar to biological nervous systems and is capable of intelligently processing simulated information. Currently, ANNs are used for the diagnostics and monitoring of shafts in turbines [49]. The benefits of the use of neural networks can exceed many times the work required for diagnostics of the foundation for turbogenerators implemented to date.

## 6. Conclusions

The turbine set has a value that is on average 20 times higher than the cost of its foundation. The foundation, together with the turbine set and the adjacent devices, must ensure safe use in continuous operation conditions, where the turbine set shaft rotates at 3000/3600 rpm (which corresponds to a frequency of 50/60 Hz). During operation, the dynamic condition of the turbine set usually deteriorates as a result of the ongoing wear and tear processes. Changes in harmonic values in the analysis of the vibrations’ spectrum (e.g., fast Fourier transformation) affect the harmonically variable load in both the horizontal and vertical directions. The main design goal for a machine’s foundation is to limit its movement to amplitudes that do not endanger the proper operation of the machine. In the case of high-speed machines, it is desirable to design the foundation to be low tuned, with the value of the vertical natural frequency below the operating speed of the machine.

The article presents the dynamic analysis and diagnostics of reinforced concrete foundations for machines, which are very important in terms of ensuring the proper safety, reliability, and durability of these very expensive machines. The design of a frame foundation for a turbogenerator is the most difficult task when compared to designing any other foundation. There are many parameters that influence the foundation’s response. The rigidity of the frame structure plays a key role. The individual vibration characteristics of individual elements, such as columns and beams, are very important in determining the behaviour of the foundation [50].

A real foundation of a turbogenerator in a CHP plant is presented as the case study. The dynamic analytical and experimental analysis method presented in the paper turned out to be a good tool to verify the foundation structure, making it possible to perform a sensitivity analysis of the impact of changes in various parameters. A numerical analysis (AxisVM software) was carried out using experimental data in which the bearing capacity of the foundation was determined and the natural frequencies and maximum amplitude were checked. In the analysis process, the auxiliary material tests were also very important. The experimental material tests performed were related to the strength of the concrete and the identification of reinforcement. The numerical analysis was positively verified using experimental tests. Comparison of analytical and experimental results allows optimising the calculation model of the foundation structure, as well as determining the dynamic parameters of the existing foundation structure. It also enables the behaviour of the foundation after reconstruction and strengthening of its structure, as well as the damage or remaining service time to be determined. A key factor in the successful design of the foundation of a turbogenerator is a precise engineering analysis of the foundation response to dynamic loads caused by the machine operation.

## Figures and Tables

**Table 1:** Results of the vibration acceleration measurement, vibration velocity, and foundation displacement (average value).

Measurement No.	Amplitude Spectrum RMS m/s^2^			Amplitude Spectrum RMS mm/s			Amplitude Spectrum RMS mm		
	X	Y	Z	X	Y	Z	X	Y	Z
1	0.027–24	0.08	0.061	0.18	0.57	0.4	0.002	0.046	0.003
2	0.016–25	0.0058	0.05	0.11–12	0.032	0.4	0.002	0.0024	0.005
3	0.014–32	0.019	0.003	0.11	0.10	0.09	0.002	0.002	0.005
4	0.014–50	0.063	0.03	0.05	0.085	0.095	0.001	0.005	0.002
5	0.006–50	0.003	0.006	0.04	0.025	0.02	0.002	0.001	0.001
6	0.0022–18	0.0045	0.016	0.03	0.03	0.09	0.002	0.002	0.005
7	0.038–50	0.025–23	0.04	0.11	0.16	0.25–25	0.004	0.002	0.004
8	0.017–22	0.022	0.35–17	0.12	0.15	0.33–18	0.002	0.001	0.003

**Table 2:** Summary of concrete the results of compressive strength tests.

Localisation	Measurement Point	Drilling Diameter [mm]	Compressive Strength [MPa]
Foundation	O1 (F1)	100	34.00
	O2 (F2)	100	34.60
	O3 (F3)	100	43.20
	O4 (F4)	100	37.50
	O5 (F5)	100	32.20
	O6 (F6)	100	29.50
	O7 (F7)	100	34.40

**Table 3:** Summary of sclerometer test results for the horizontal beam.

Site	Angle	Readings	*L_i_*	*L_i_ − L_av_*	(*L_i_ − L_av_*)^2^
	[Deg]				
1	90	45 44 47 47 44 44 46 46 47	45.6	−2.67	7.11
2	90	52 52 49 49 51 50 49 49 49	50	1.78	3.16
3	90	48 47 47 48 47 48 48 49 49	47.9	−0.33	0.11
4	90	50 52 52 49 49 50 52 52 52	50.9	2.67	7.11
5	90	44 44 46 46 48 46 47 48 48	46.3	−1.89	3.57
6	90	48 49 50 49 48 48 48 49 49	48.7	0.44	0.2

*n* = 6—number of readings.

**Table 4:** Summary of sclerometer test results for the middle column.

Site	Angle	Readings	*L_i_*	*L_i_ − L_av_*	(*L_i_ − L_av_*)^2^
	[Deg]				
1	90	51 51 50 52 50 50 51 50 52	50.8	−1.35	1.83
2	90	52 52 52 50 51 52 52 50 52	51.4	−0.69	0.47
3	90	51 52 54 54 52 54 52 52 52	52.6	0.43	0.18
4	90	54 52 54 54 52 53 53 54 52	53.1	0.98	0.96
5	90	54 54 52 52 52 52 54 53 53	52.9	0.76	0.58
6	90	52 52 52 52 54 51 52 51 52	52	−0.13	0.02

*n* = 6—number of readings.

## Data Availability

Not applicable.
